# Gut microbiota and autism spectrum disorders: where do we stand?

**DOI:** 10.1186/s13099-023-00575-8

**Published:** 2023-10-25

**Authors:** Sa’ed H. Zyoud, Muna Shakhshir, Amani S. Abushanab, Amer Koni, Moyad Shahwan, Ammar A. Jairoun, Adham Abu Taha, Samah W. Al-Jabi

**Affiliations:** 1 Department of Clinical and Community Pharmacy, College of Medicine and Health Sciences, An-Najah National University, 44839 Nablus, Palestine; 2 Clinical Research Centre, An-Najah National University Hospital, 44839 Nablus, Palestine; 3 Department of Nutrition, An-Najah National University Hospital, 44839 Nablus, Palestine; 4 Division of Clinical Pharmacy, Hematology and Oncology Pharmacy Department, An- Najah National University Hospital, 44839 Nablus, Palestine; 5 College of Pharmacy and Health Sciences, Ajman University, Ajman, United Arab Emirates; 6 Health and Safety Department, Dubai Municipality, Dubai, United Arab Emirates; 7 Department of Biomedical Sciences, Faculty of Medicine and Health Sciences, An-Najah National University, 44839 Nablus, Palestine; 8 Department of Pathology, An-Najah National University Hospital, 44839 Nablus, Palestine

**Keywords:** ASD, Autism, Gut microbiota, Autism spectrum disorders, Bibliometric, Scopus, *Reference citation analysis*

## Abstract

**Background:**

Children with autism spectrum disorder (ASD) often have digestive problems and microbial imbalances in their guts, suggesting that these conditions may play a role in the development of the disorder. Scopus-based research on the gut microbiota and ASD was examined in this bibliometric analysis to shed light on the current state of research and identify potential hotspots for future work in this area.

**Methods:**

We searched documents from the Scopus database and *reference citation analysis* to collect published data on the gut microbiota and ASD from 2003 to 2022. The downloaded document records were exported to VOSviewer v.1.6.19 to examine and visualize the collaboration between countries and determine the research hotspots.

**Results:**

The search yielded 958 articles specifically dedicated to gut microbiota and ASD. The number of publications in this field increased rapidly after 2013, with a peak in 2022. The United States (*n* = 267; 27.87%) was the most active country, followed by China (*n* = 171; 17.85%) and Italy (n = 96; 10.02). International collaboration was observed, with the USA playing a central role. *University College Cork*, Ireland, was the most productive institution (*n =* 24; 2.51%). The *National Natural Science Foundation of China* was the most active funding agency (*n* = 76; 7.93%). *Nutrients* journal had the highest number of publications (*n* = 28; 2.92%). The articles related to gut microbiota and ASD were highly cited, with an *h*-index of 108. The research themes identified focused on the modulation of gut microbiota as a potential therapy for children with ASD and gut-brain axis dysfunction in ASD.

**Conclusions:**

In recent years, the study of gut microbiota and its association with ASD has garnered considerable interest as an emergent field of study. The results of this study substantially enhance our current understanding of the knowledge landscape in this field and illuminate potential avenues for future research. It is essential to emphasize the significance of devoting more resources to the newest and most promising research areas, such as investigating the potential therapeutic benefits of modulating the intestinal microbiota in children with ASD. This research has enormous potential and merits intensified focus and investigation.

## Background

Autism spectrum disorder (ASD) is an umbrella term that includes various conditions with a wide range of origins [1, 2]. Notably, the prevalence of ASD has steadily increased over the past decade. This upward trend can be attributed to increased awareness among healthcare and educational personnel and modifications in diagnostic criteria [3, 4]. Emerging evidence also links ASD to a number of environmental factors, such as dietary factors, maternal infections, intestinal dysbiosis, stress, medications, pesticide exposure, and antibiotic use during pregnancy [5–10]. Examining these environmental connections [5] can aid in the comprehension of the increase in ASD occurrence. Given the prevalence of ASD among children and adolescents, which ranges from 0.6 to 1.7% [11–13], it poses a significant public health concern.

The composition of the gut microbiota is thought to play a crucial role in human health and disease, influencing immunological development, physiological homeostasis, amino acid metabolism, glutathione metabolism, and other processes. Consequently, the involvement of the gut-brain axis in ASD becomes evident [14]. Recent scientific interest has been directed toward exploring the potential contribution of gut microbiota composition as a cofactor in ASD development, given the established bidirectional communication between the gut and the brain, known as the “gut-brain axis” [3, 15]. To inform public policy, promote awareness, and define research objectives, it is critical to have global estimates of research related to gut microbiota and ASD.

Several studies have evaluated research trends in the fields of microbiota [16–27] and ASD [28–33] separately. These studies used bibliometric analysis to track and measure research activity and its development over time. Bibliometric analysis is often used to evaluate publication output and assess the amount of research in a specific field [34–38]. Until now, no previous study in the literature has been a bibliometric analysis of research related to gut microbiota composition and ASD. The growing interest in the gut-brain axis and its potential involvement in ASD has resulted in a notable increase in scientific investigations focusing on gut microbiota diversity in individuals with ASD. To enhance comprehension of the worldwide research landscape pertaining to this particular subject, a bibliometric study was performed on publications covering the last two decades. The study conducted an analysis of publication patterns, revealing notable trends such as substantial annual growth in the volume of published papers, expanding global collaboration, and the identification of prominent research domains within this exciting science. The investigation additionally unveiled the prominent countries, institutions, funding agencies, journals, and publications inside the domain. The current study offers a significant contribution to the body of research pertaining to the relationship between gut microbiota and ASD, hence serving as a valuable resource for researchers with an interest in this area. Identifying research gaps, providing guidance for future research areas, and helping develop new solutions are potential benefits that can be derived from this approach.

## Methods

### Database used

In the present investigation, we collected scientific literature related to gut microbiota and ASD from Scopus, a comprehensive academic database developed by Elsevier. Scopus boasts an extensive collection of over 25,000 indexed journals spanning various scientific disciplines. It offers valuable features for evaluating research trends and growth and facilitates data export to other software for literature mapping purposes [39]. When examining research trends and patterns, most studies preferentially employ Scopus, Web of Science, or PubMed to access global scientific literature [40–42]. Scopus’s broader inclusivity compared to Web of Science or PubMed makes it a favored choice [43]. Furthermore, Scopus is accessible at no cost to many scholars in low-income countries through the HINARI initiative [44]. A comparative analysis revealed that approximately 99.11% of the journals indexed in Web of Science are also available in Scopus [45]. In addition, Scopus allows researchers to export and thoroughly examine the obtained data, facilitating activities such as data mapping and statistical analysis [41, 46–50]. Finally, it is crucial to emphasize that Scopus is widely regarded as the primary database utilized for conducting bibliometric studies and accessing articles across various scientific disciplines [17, 20, 51–54]. All data were obtained from Scopus on 25 May 2023. Scopus is the largest peer literature abstract and citation database that helped researchers harvest the largest number of publications [55, 56] related to bibliometric applications in research related to gut microbiota composition and ASD.

### Search strategy

We searched Scopus for documents that included at least one of the following keywords in their titles and/or abstracts: “autism”, “autistic”, “Asperger syndrome”, “pervasive developmental disorder”, “PDDNOS”, and “Gastrointestinal Microbiomes” OR “Gut Microbiome” OR “Gut Microflora” OR “Gut Microbiota” OR “Gastrointestinal Flora” OR “Gut Flora” OR “Gastrointestinal Microbiota” OR “Gastrointestinal Microbial Community” OR “Gastrointestinal Microflora” OR “Gastric Microbiome” OR “Gastric Microbiota” OR “Gastric flora” OR “Gastric Microflora” OR “Intestinal Microbiome” OR “Intestinal Microbiota” OR “Intestinal Microflora” OR “Intestinal Flora” OR “Enteric Bacteria” OR “Enteric microbiota” OR “Enteric flora” OR “Enteric microflora” OR “Enteric microbiome” OR “Digestive microbiota” OR “Digestive flora” OR “Digestive microflora” OR “Digestive microbiome” OR “Fecal microbiota” OR “Fecal flora” OR “Fecal microflora” OR “Fecal microbiome” OR “Faecal microbiota” OR “Faecal flora” OR “Faecal microflora” OR “Faecal microbiome” OR “Colonic microbiota” OR “Colonic flora” OR “Colonic microflora” OR “Colonic microbiome” OR “probiotic” OR “Bifidobacterium” OR “Saccharomyces” OR “*Escherichia coli”* OR “Lactobacillus” OR “dysbiosis”).

Keywords were selected according to the search within Medical Subject Headings (MeSH) in PubMed or from previous bibliometric studies [57–62] or meta-analysis and systematic studies [3, 63–67]. The results were filtered by limiting documents to journal research, while proceedings, conferences, books, and book chapters were excluded. We selected the period between 2003 and 2022 to examine recent trends. Search query used for data extraction from Scopus looked like this: ((TITLE-ABS(autism) OR TITLE-ABS(autistic) OR TITLE-ABS(ASD) OR TITLE-ABS(Asperger syndrome) OR TITLE-ABS(pervasive developmental disorder) OR TITLE-ABS(PDDNOS)) AND (TITLE-ABS(“Gastrointestinal Microbiomes” OR “Gut Microbiome” OR “Gut Microflora” OR “Gut Microbiota” OR “Gastrointestinal Flora” OR “Gut Flora” OR “Gastrointestinal Microbiota” OR “Gastrointestinal Microbial Community” OR “Gastrointestinal Microflora” OR “Gastric Microbiome” OR “Gastric Microbiota” OR “Gastric flora” OR “Gastric Microflora” OR “Intestinal Microbiome” OR “Intestinal Microbiota” OR “Intestinal Microflora” OR “Intestinal Flora” OR “Enteric Bacteria” OR “Enteric microbiota” OR “Enteric flora” OR “Enteric microflora” OR “Enteric microbiome” OR “Digestive microbiota” OR “Digestive flora” OR “Digestive microflora” OR “Digestive microbiome” OR “Faecal microbiota” OR “Faecal flora” OR “Faecal microflora” OR “Faecal microbiome” OR “Fecal microbiota” OR “Fecal flora” OR “Fecal microflora” OR “Fecal microbiome” OR “Colonic microbiota” OR “Colonic flora” OR “Colonic microflora” OR “Colonic microbiome” OR “probiotic” OR “Bifidobacterium” OR “Saccharomyces” OR “*Escherichia coli*” OR “Lactobacillus” OR “dysbiosis”)) AND PUBYEAR > 2002 AND PUBYEAR < 2023) AND (LIMIT-TO (SRCTYPE,“j”)) AND (EXCLUDE (DOCTYPE,“er”)).

In each step of the search query, quotation marks were utilized to precisely retrieve the exact phrase. Additionally, the asterisk truncation served as a versatile wild card, allowing the retrieval of any potential term.

### Validation of search strategy

The search was limited to the title and/or abstract of the publications in the Scopus database to achieve reasonable accuracy of the collected data because if it was expanded to all search fields, several publications would be included that are not about gut microbiota and ASD (i.e., false-positive data). This approach would substantially increase specificity while acknowledging a slight reduction in sensitivity [19, 68–70]. The adopted research strategy underwent thorough validation to ensure the absence of false-positive results. Initial validation involved reviewing the titles and abstracts of documents with even numbers (10, 20, 30, 40, etc.) until the end, carefully excluding any false-positive findings. This process of fine-tuning and exclusion continued until all randomly screened results were completely free from false positives.

The research productivity of ten active authors in the field was investigated to validate the research strategy against false-negative results (missing findings). A comparison was made between the numbers obtained through their research output and the results obtained using the research strategy. This assessment was conducted using the Spearman correlation test. The findings revealed a significant and robust correlation (p < 0.001; r = 0.983), indicating a high level of validity for the research strategy. Sweileh et al. [41, 71, 72] adopted this validation approach, which proved to be effective.

### Bibliometric analysis

The following indicators were selected and analysed: document type, number of articles published per year, top 10 countries contributing to publications, top 10 productive institutions, top 10 funding agencies, top 10 journals with their impact factors (IF), and top 20 cited articles. Ranked order frequencies of publications were used to identify the leading countries, institutions, funding agencies, journals, and the most influential publications in the field. IF was extracted from Clarivate Analytics in 2020, Journal Cited Reports (JCR). The data from *reference citation analysis* (RCA) are utilized in the calculation of the *impact index per article* for the top ten publications with the highest number of citations. Baishideng Publishing Group Inc., headquartered in Pleasanton, CA 94,566, USA, is the proud owner of RCA, an open and comprehensive citation analysis database that spans various disciplines [73–75].

### Visualization analysis

The downloaded document records were exported to VOSviewer v.1.6.19 (https://www.vosviewer.com/) to examine and visualize the collaboration between countries and determine research hotspots. VOSviewer represented terms in both titles and abstracts by colors and size of the circles according to the term occurrence. Hotspots are defined as terms and their frequency in common science fields [76, 77]. VOSviewer grouped terms into different clusters based on the co-occurrence analysis results. The cocitation analysis of countries was presented as a network visualization map, with the division of different clusters identified as related research fields and their frequency of occurrence calculated.

## Results

### General characteristics of the retrieved articles

A comprehensive search at the global level from 2003 to 2022 yielded a remarkable collection of 390,361 documents pertaining to gut microbiota mentioned in titles and abstracts. Among these scholarly publications, 958 were specifically dedicated to the exploration of gut microbiota and its connection to ASD (autism spectrum disorder). Delving deeper, this body of work comprised 580 articles (60.54%), 354 reviews (36.95%), and 24 miscellaneous entries (2.51%), including letters and editorials. A significant and remarkable positive correlation with a correlation coefficient of 0.984 (p < 0.001) was identified between the quantity of publications related to reviews and the quantity of publications related to original articles concerning time.

### Evolution and growth of publications

**Fig. 1:**
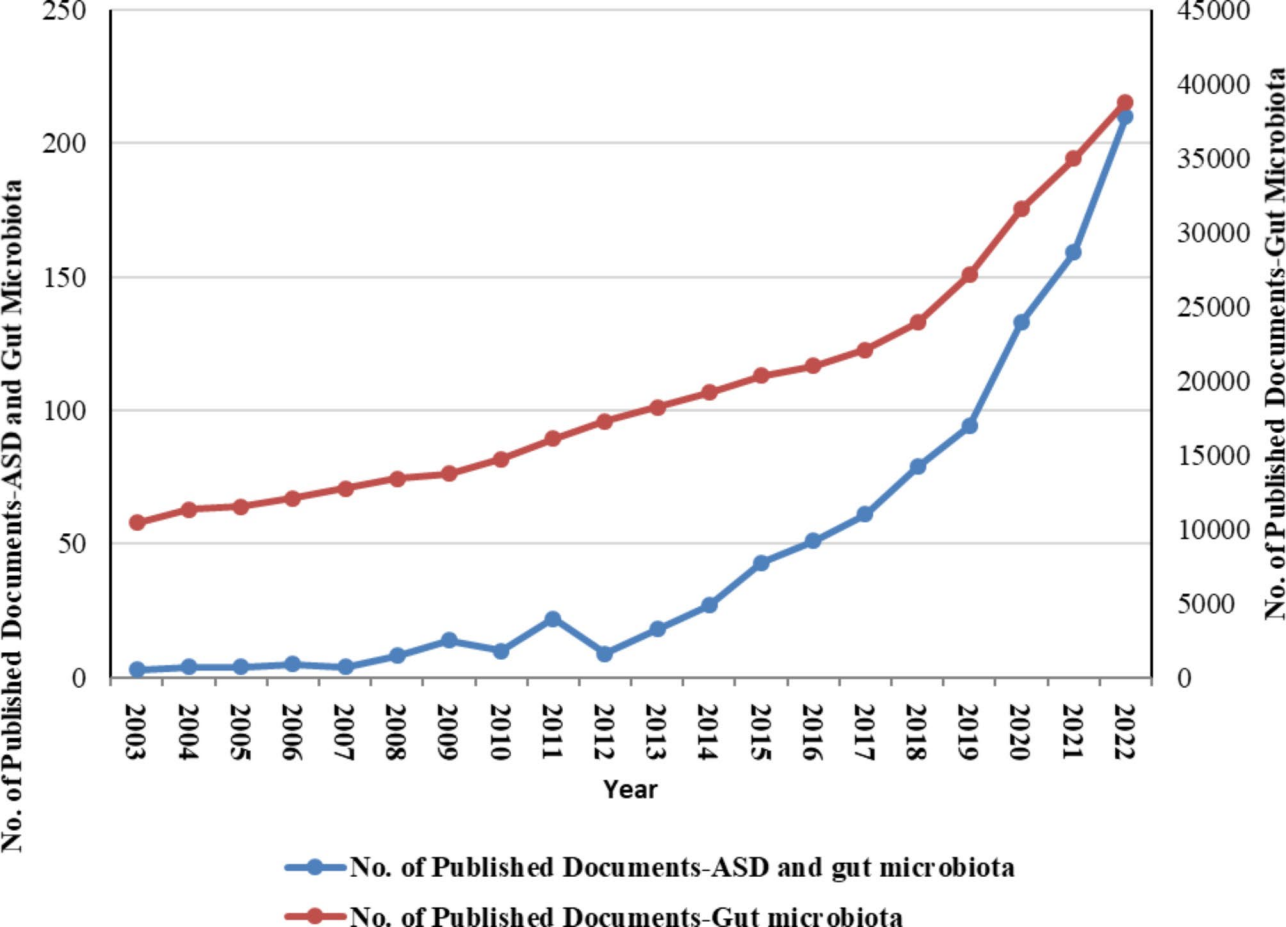
The global number of publications related to gut microbiota and autism spectrum disorders from 2003 to 2022

The trends in the number of publications found by Scopus related to the gut microbiota and ASD over the past 20 years are shown in Fig. 1. A significant and robust positive correlation was observed between publication productivity concerning gut microbiota across various fields and productivity specifically related to gut microbiota and ASD (*r* = 0.973, *P* < 0.001). The number of publications increased slightly between 2003 and 2013, with fewer than 9.18 publications annually. However, after 2013, the number of published articles in this field increased rapidly, with an average of more than 95 published articles per year, and peaked in 2022, with 210 publications.

### Top ten active countries

Researchers from 74 countries have signed all included publications related to gut microbiota and ASD. Publications from the top 10 countries represented 74.2% of all literature on gut microbiota and ASD (Table 1). Two hundred and sixty-seven contributions came from the USA, followed by 171 from China and 96 from Italy.

**Table 1:** Top 10 countries most productive in terms of relevant articles related to gut microbiota and autism spectrum disorders from 2003 to 2022

Ranking	Country	No. of documents	% ^a^
1st	United States	267	27.87
2nd	China	171	17.85
3rd	Italy	96	10.02
4th	Canada	52	5.43
5th	United Kingdom	49	5.11
6th	Spain	40	4.18
7th	South Korea	37	3.86
8th	Australia	35	3.65
9th	Poland	34	3.55
10th	Saudi Arabia	33	3.44

^a^ The variance between the 74.2% mentioned in the text and the sum of 84.96% in Table 1 can be attributed to the phenomenon of overlapping research productivity among countries. This overlap occurs when researchers from different countries collaborate on research projects or when the same research output is attributed to multiple countries

### International research collaboration

**Fig. 2:**
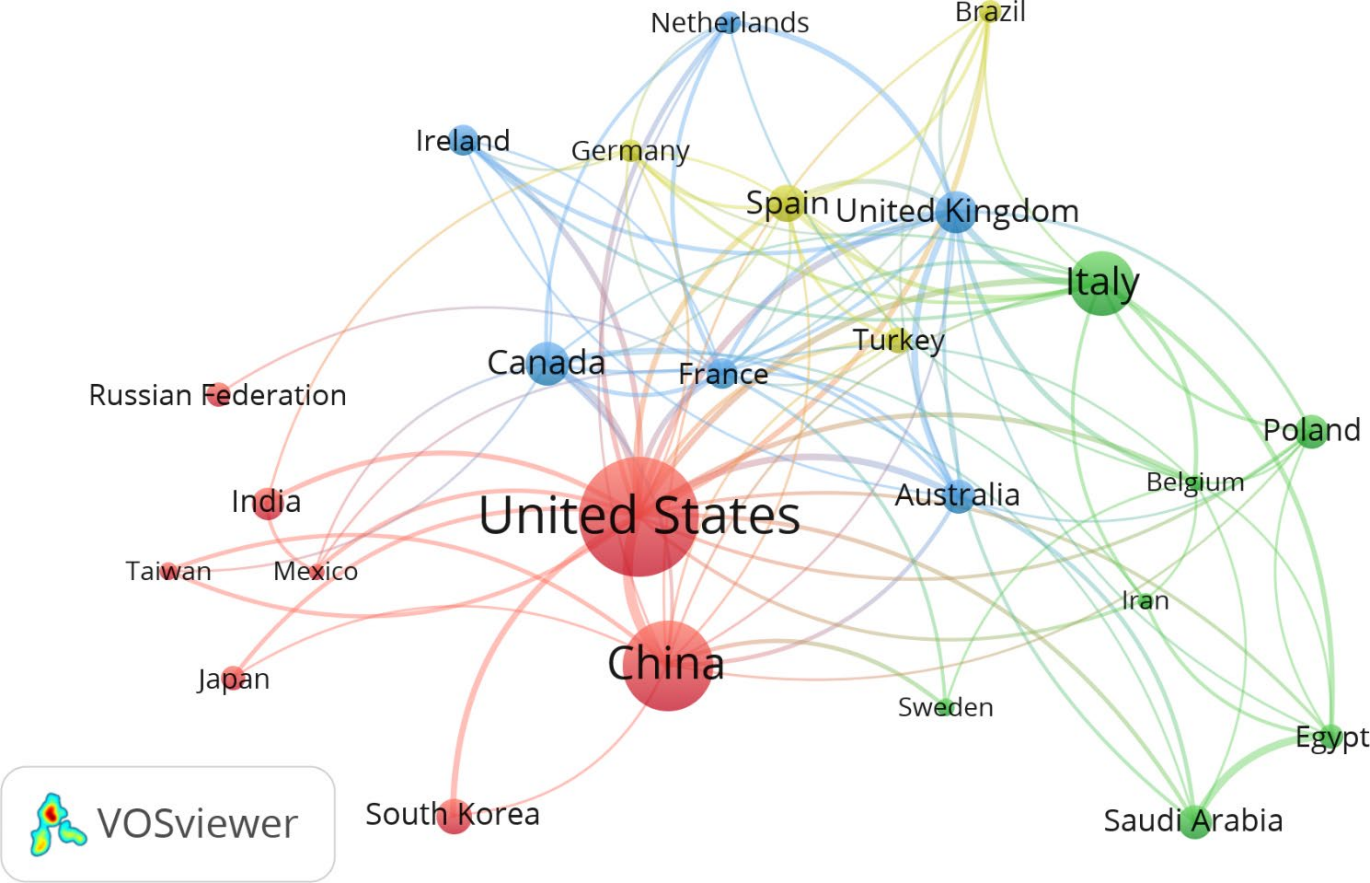
Visualization map of international collaboration in countries with a minimum productivity of 10 publications

For countries with a minimum of ten publications, international collaboration was visualized for relevant research related to gut microbiota and ASD between 2003 and 2022 (Fig. 2). The map illustrates that the USA is at the center of this area with many linking lines, indicating a significant number of partner countries with the USA.

### Top ten active institutions

The ten most productive institutes for relevant articles related to gut microbiota and ASD from 2003 to 2023 are listed in Table 2. Among these institutions, the top ten collectively contributed to 15.45% (n = 148) of the published articles. *University College Cork*, Ireland, was the most productive institution (*n =* 24; 2.51%), followed by *APC Microbiome Ireland*, Ireland (*n* = 23; 2.4%), *King Saud University*, Saudi Arabia (*n* = 23; 2.4%) and others. There were five institutes from the United States, followed by two from Ireland. Meanwhile, one institution each for Saudi Arabia, China, and Italy was on the list. The publications of the top 10 institutes accounted for 15.45% of all literature related to gut microbiota and ASD.

**Table 2:** Top 10 productive institutes in research related to gut microbiota and autism spectrum disorders from 2003 to 2022

Ranking ^a^	Institute	Country	No. of documents	% ^b^
1st	*University College Cork*	Ireland	24	2.51
2nd	*APC Microbiome Ireland*	Ireland	23	2.40
2nd	*King Saud University*	Saudi Arabia	23	2.40
4th	*Ministry of Education China*	China	22	2.30
4th	*Massachusetts General Hospital*	USA	22	2.30
6th	*Harvard Medical School*	USA	20	2.09
6th	*College of Sciences*	USA	20	2.09
8th	*Consiglio Nazionale delle Ricerche*	Italy	19	1.98
9th	*Arizona State University*	USA	18	1.88
10th	*University of California, Los Angeles*	USA	15	1.57

^a^ Gap is left in the next ranking number when specific institutes are given the same number

^b^ The variance between the 15.45% mentioned in the text and the sum of 21.52% in Table 2 can be attributed to overlapping research productivity among institutions. This overlap occurs when researchers from different institutions collaborate on research projects, resulting in the same research output being attributed to multiple institutions

### Top ten funding agencies

A total of 502 (52.4%) publications were funded projects of the retrieved articles. The top 10 funding agencies for relevant articles related to gut microbiota and ASD from 2003 to 2022 are listed in Table 3. Among these funding agencies, the leading 10 collectively contributed to 23.90% (*n* = 229) of the total published articles. The *National Natural Science Foundation of China* (*n* = 76; 7.93%) was the most active funding agency in this field, followed by the *National Institutes of Health*, USA (*n* = 63; 6.58%) and *National Institute of General Medical Sciences*, USA (*n* = 23; 2.4%).

**Table 3:** Top ten productive funding agencies in research related to gut microbiota and autism spectrum disorders from 2003 to 2022

Ranking ^a^	Funding agency	Country	No. of documents	%
1st	*National Natural Science Foundation of China*	China	76	7.93
2nd	*National Institutes of Health*	USA	63	6.58
3rd	*National Institute of General Medical Sciences*	USA	23	2.40
4th	*National Institute of Mental Health*	USA	20	2.09
5th	*Autism Research Institute*	USA	19	1.98
5th	*National Institute of Allergy and Infectious Diseases*	USA	19	1.98
7th	*National Institute of Diabetes and Digestive and Kidney Diseases*	USA	16	1.67
7th	*National Research Foundation of Korea*	South Korea	16	1.67
9th	*Natural Sciences and Engineering Research Council of Canada*	Canada	13	1.36
9th	*Science Foundation Ireland*	Ireland	13	1.36

^a^ Gap is left in the next ranking number when funding agencies are given the same number

^b^ The variance between the 23.90% mentioned in the text and the sum of 29.02% in Table 3 can be attributed to overlapping research productivity among funding agencies. In several instances, the same research projects may have received financial support from multiple funding agencies, resulting in the same research output being attributed to multiple funding agencies

### Top ten active journals

As shown in Table 4, the top 10 journals/source titles account for approximately 16.48% of the total publications on research related to gut microbiota and ASD. *Nutrients* (IF 6.706, 2022) had the highest number of publications, with 28 publications.

**Table 4:** Top 10 productive journals/source titles in research related to gut microbiota and autism spectrum disorders from 2003 to 2022

Ranking ^a^	Journal/source title	No. of documents	%	IF ^b^
1st	*Nutrients*	28	2.92	6.706
2nd	*International Journal of Molecular Sciences*	22	2.30	6.208
3rd	*Scientific Reports*	18	1.88	4.996
4th	*Brain Behavior and Immunity*	14	1.46	19.227
4th	*Frontiers in Psychiatry*	14	1.46	5.435
4th	*Plos One*	14	1.46	3.752
7th	*Frontiers in Cellular and Infection Microbiology*	12	1.25	6.073
7th	*Frontiers in Microbiology*	12	1.25	6.064
7th	*Frontiers in Neuroscience*	12	1.25	4.677
7th	*Microorganisms*	12	1.25	4.926

^a^ Gap is left in the next ranking number when specific journals are given the same number

^b^ Impact factors (IF) based on Clarivate Analytics Journal Citation Reports (JCR) 2022

### Citation analysis

All articles related to the gut microbiota and ASD have been cited 50,845 times since 2003, with an *h*-index of 108. The top 10 most highly cited articles are shown in Table 5. Six documents were published after 2015 [78–87]. The citations varied significantly, ranging from 679 to 2132, demonstrating the diverse attention garnered by the articles. Notably, the top ten most cited articles exhibited a remarkable *impact index per article*, ranging from 94.8 to 289.0, highlighting their influential contributions to the field. (Table 5).

**Table 5:** The 10 most cited publications in research related to gut microbiota and autism spectrum disorders from 2003 to 2022

Ranking	Authors	Title	Year	Source Title	Cited by	Impact Index Per Article a
1st	Hsiao et al. [82]	“Microbiota modulate behavioral and physiological abnormalities associated with neurodevelopmental disorders”	2013	*Cell*	2132	208.4
2nd	Cryan et al. [80]	“The microbiota-gut-brain axis”	2019	*Physiological Reviews*	1509	289.0
3rd	Carabotti et al. [79]	“The gut-brain axis: Interactions between enteric microbiota, central and enteric nervous systems”	2015	*Annals of Gastroenterology*	1364	166.3
4th	Fung et al. [81]	“Interactions between the microbiota, immune and nervous systems in health and disease”	2017	*Nature Neuroscience*	998	163.3
5th	Mayer et al. [84]	“Gut/brain axis and the microbiota”	2015	*Journal of Clinical Investigation*	869	96.8
6th	Rivière et al. [86]	“Bifidobacteria and butyrate-producing colon bacteria: Importance and strategies for their stimulation in the human gut”	2016	*Frontiers in Microbiology*	841	106.3
7th	Nguyen et al. [85]	“How informative is the mouse for human gut microbiota research?”	2015	*DMM Disease Models and Mechanisms*	771	94.8
8th	Sharon et al. [87]	“The Central Nervous System and the Gut Microbiome”	2016	*Cell*	769	116.2
9th	Kang et al. [83]	“Microbiota Transfer Therapy alters gut ecosystem and improves gastrointestinal and autism symptoms: An open-label study”	2017	*Microbiome*	736	122.5
10th	Buffington et al. [78]	“Microbial Reconstitution Reverses Maternal Diet-Induced Social and Synaptic Deficits in Offspring”	2016	*Cell*	679	106.0

^a^*The impact index per article* is presented based on reference citation analysis (Source: Baishideng Publishing Group Inc. (Pleasanton, CA 94,566, USA))

### Research themes

**Fig. 3:**
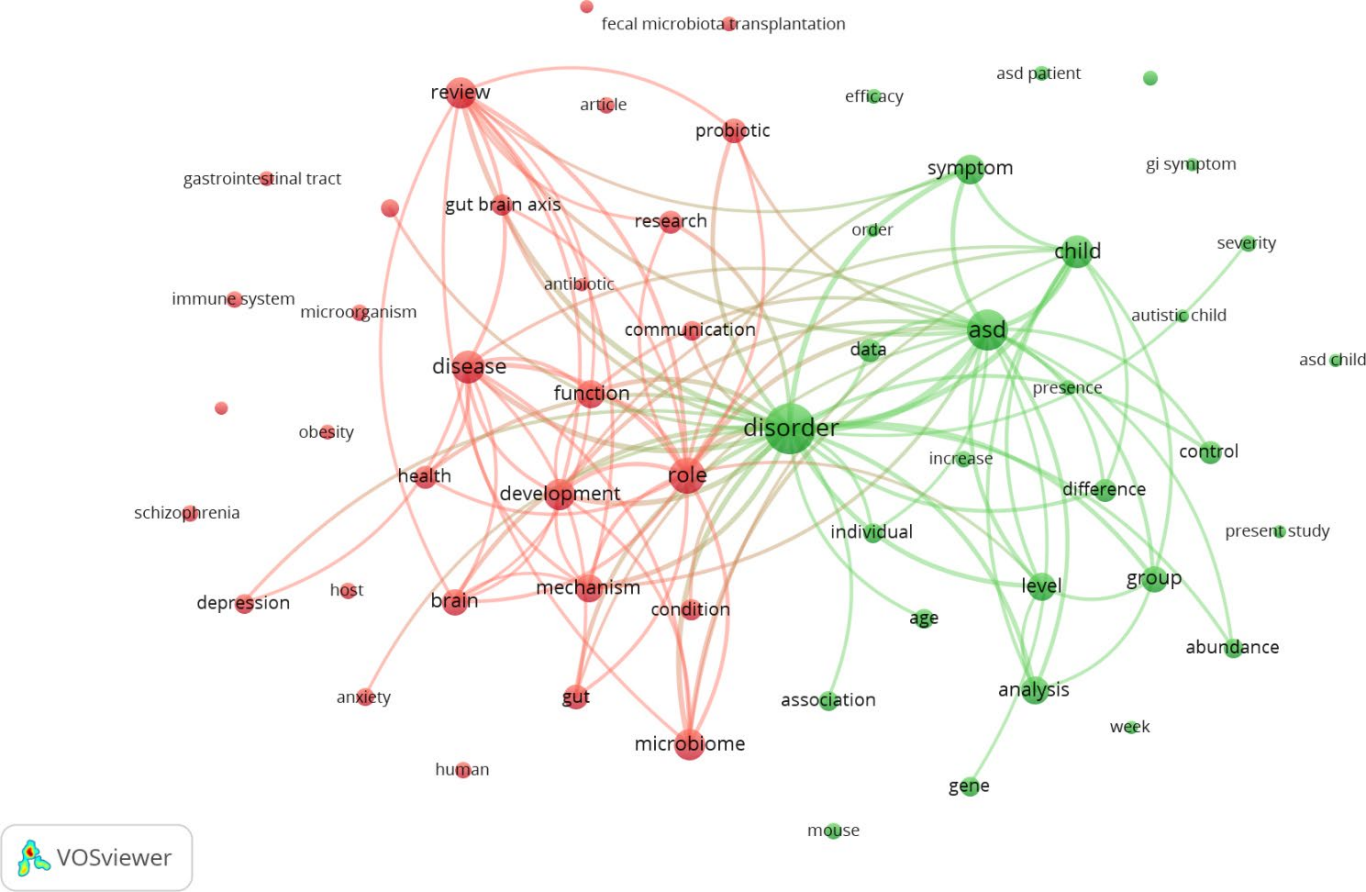
Research topics clustered by mapping of co-occurrences of terms in the title/abstract for publications pertaining to gut microbiota and autism spectrum disorders from 2003 to 2022

The terms of the 958 publications were analyzed in the software VOSviewer 1.6.9. Term co-occurrence network maps were developed to establish the hot topics within the research area (Fig. 3). The selection criteria were terms that appeared in the titles and abstracts more than 50 times in the selected publications. Of the 20,013 terms used, 96 terms were divided into two main clusters: red and green. As illustrated in Fig. 3, the two main clusters were “modulation of the gut microbiota is a potential therapy for children with ASD” (green cluster) and “microbiota and gut-brain axis dysfunction in ASD” (red cluster).

## Discussion

This is the first study to provide a detailed bibliometric analysis of worldwide research output in the field of gut microbiota composition and ASD. In the current study, we analyze research in the field of gut microbiota and ASD from various aspects, such as document type, the number of articles published per year, the top 10 countries that contributed to publications, the top 10 productive institutions, the top 10 journals with their impact factors (IF), the top ten cited articles and co-occurrence analysis of the most commonly used terms, to identify the most researched topics relevant to the field of gut microbiota composition and ASD.

The current study also showed that more than 90% of publications in the field of gut microbiota composition and ASD had been published over the last decade. The increase in global research production in the field of gut microbiota composition and ASD may be related to an emerging problem that has demonstrated a link between gut microbiota composition and ASD as an increase in the number of publications in this field [88–91].

Since 2013, there has been a significant rise in research output regarding the microbiome-gut-brain axis (MGBA), paralleling the overall increase in microbiome-related studies [19, 92–94]. Numerous other bibliometric investigations [18, 22, 23, 95–97] have reported similar findings. A potential explanation for this surge in publications can be traced back to the second phase of the Integrative Human Microbiome Project (iHMP), initiated by the National Institutes of Health (NIH) in 2013 [98]. The number of articles dedicated to MGBA research experienced a rapid upturn between 2009 and 2018. This upward trend can be attributed to the growing interest among experts in psychiatry, neurology, and gastroenterology, such as Cryan J.F., Dinan T.G., Clarke G., Bienenstock J., Forsythe P., Stanton C., Quigley E.M.M., Bercik P., O’Mahony S.M., Shanahan F., Foster J.A., Moloney R.D., and others. These professionals have delved into the physiological role of the gut microbiota in brain function and behavior, recognizing it as a promising avenue for therapeutic interventions in various diseases [19].

Of the prolific countries, the US ranked first regarding the number of articles, collaboration with other countries, and citation patterns. Such findings are consistent with earlier bibliometric studies [70, 99–102]. The emergence of these results was expected, owing to the United States’ reputation for hosting a wide array of exceptional international journals and accomplished scientists across diverse scientific domains [103]. Additional factors that likely contributed to these outcomes encompass the comparatively substantial research funding allocated within the USA and its rapid economic advancement [104]. Furthermore, due to many institutions and authors in the USA, the number of collaborations with other international countries could be expanded and increased [105].

A remarkable finding highlighting the organization’s commitment to advancing research in this field is the prominent role of the *National Natural Science Foundation of China* (NSFC) as a funding agency for studies related to gut microbiota and ASD. This finding carries great significance, as it underscores the organization’s dedication to pushing the boundaries of research. China has made substantial investments in establishing state-of-the-art laboratories and research institutes [106, 107], which have attracted researchers interested in exploring the link between gut microbiota and autism spectrum disorder. The provision of exceptional research facilities may even enhance the chances of securing funding from the NSFC, solidifying its position as a leading supporter of research in this field. The alignment between the goals of the NSFC and the significance of studying gut microbiota in relation to ASD is evident. Considering the mounting evidence linking gut microbiota to neurodevelopmental issues, the NSFC may have recognized the importance of expanding scientific knowledge in this domain and potentially uncovering novel therapeutic approaches for ASD. Consequently, they may have allocated a substantial budget to support research in this area. Moreover, the surge in Chinese publication numbers can be attributed, in part, to the establishment of the NSFC’s fund dedicated to investigating the significance and mechanisms of the ‘gut-liver axis’ in gastrointestinal diseases (H03) [108]. This research initiative focuses on comprehensively studying the gut microbiome, metabolites, nutrition, neurotransmitters, gastrointestinal hormones, and bile acids, specifically emphasizing their intricate interactions with the gastrointestinal tract or liver-localized immune cells/factors contributing to various functional roles. These projects aim to further our comprehension of the gut-liver axis and its important significance in advancing diagnostic and therapeutic approaches for gastrointestinal disorders [108].

The National Institutes of Health (NIH) and its associated institutes have made major contributions to the progress of research on the association between gut microbiota and ASD [109]. The fact that ASD is a complex condition influenced by several factors, including genetics, neurology, immunology, and metabolism, among others, is acknowledged in this statement [110–113]. These NIH-affiliated institutes show their commitment to thoroughly examining and addressing all potential avenues to gain understanding of and develop interventions for ASD by allocating financial resources to research in this particular area. These findings also demonstrate the NIH’s dedication to expanding the realm of knowledge. The involvement of numerous specialized institutes within the NIH indicates a coordinated effort to approach ASD from a variety of perspectives. ASD is a complex and difficult condition. Adopting an interdisciplinary approach is essential for making significant strides in the field and ultimately raising the standard of living for people with ASD [114].

The term co-occurrence network map reflects the research hotspots in the area of gut microbiota composition and ASD through the weight and strength of the spheres and words [76]. In the current study, most publications related to gut microbiota composition and ASD focused on terms relevant to the topics of “modulation of the gut microbiota composition is a potential therapy for children with ASD” and “microbiota and gut-brain axis in ASD”. Research on the gut microbiota composition and ASD has seen an increase in publications in this time period, which may be due to the many hot topics being published at the same time. These findings suggest new therapeutic and diagnostic concepts for neuropsychiatric disorders (such as depression and ASD) and neurodegenerative disorders (such as Parkinson’s disease) [115, 116]. Interest in therapies targeting the dysbiotic microbiota associated with ASD has steadily grown. Various approaches, including diet [117], probiotics [118], prebiotics [119], antibiotics [120], antifungal supplementation [121], fecal microbiota transplantation [122], and microbiota transfer therapy [123], have been proposed as potential therapeutic options for ASD. Some of these interventions have already shown promise in treating certain psychiatric disorders, such as depression or anxiety. However, in regard to ASD, the available evidence for these interventions is limited [91, 124]. Initial evidence indicates that therapies using microbiota have the potential to bring about beneficial effects for individuals diagnosed with ASD. However, it is of utmost importance to undertake meticulously planned, extensive randomized controlled trials on a large scale, employing standardized protocols. These trials aim to establish the efficacy and safety of these treatments [91, 124]. Numerous studies have extensively investigated the connection between different gut microbiota and ASD. Nonetheless, identifying the exact patterns of alterations in microbial profiles among children with ASD has proved to be challenging. A comprehensive meta-analysis now sheds light on this matter by synthesizing the findings from 26 studies, predominantly published between 2010 and 2023 [125]. The investigations primarily took place in China, the United States, Australia, and Italy, with additional contributions from India, Japan, and Spain [125].

### Limitations

Although this is the first bibliometric analysis to assess worldwide research output in the field of gut microbiota and ASD, our study has several limitations, as indicated by previous similar studies [41, 126]. First, we solely relied on Scopus as the data source for gut microbiota and ASD research. Nonetheless, Scopus, being the largest abstract and citation database of peer literature, enabled us to access the largest number of publications. Second, we identified relevant MeSH terms related to gut microbiota and ASD in the title and abstract of the publications, potentially missing any documents that discuss these topics within their content. However, any false-negative results are unlikely to significantly impact the overall findings [41, 126]. Third, an important concern arises regarding the accuracy of affiliation information in the retrieved articles, particularly regarding the reflection of the countries where the study was conducted. This issue, commonly known as “overlap” bias, highlights the challenges when relying solely on affiliation information to determine the research’s origin. Fourth, our examination focused on gut microbiota and did not consider other factors, such as environmental or genetic influences on ASD. This limited scope may not fully represent the comprehensive research landscape. Fifth, language bias may have influenced this study, as we only included publications published in English. Last, it is important to acknowledge that this study is primarily descriptive, providing evidence of the trends and growth in this area of research. It does not evaluate the quality or relevance of the research to the field.

## Conclusions

In conclusion, this bibliometric analysis sheds light on the current research on gut microbiota and ASD. The number of publications in this field has significantly increased over the last 20 years, peaking in 2022. In terms of research output, the USA, China, and Italy are the top three nations. International collaboration is extremely important, with the USA at the center of these collaborations. Ireland’s University College Cork emerges as the institution with the highest productivity. Research in this field has received active support from funding organizations such as the National Institutes of Health in the United States and the National Natural Science Foundation of China. The main outlet for disseminating research findings in this area has been the Nutrients journal. The highly cited articles in this field also highlight the significance and influence of research on gut microbiota and ASD. The study identifies two major research themes: investigating gut-brain axis dysfunction in ASD and the modulation of gut microbiota as a potential therapy for children with ASD. The role of gut microbiota in ASD has received much attention recently, reflecting its potential importance. As we move forward, more funding and attention must be given to new research areas, with a particular emphasis on examining the therapeutic potential of altering the gut microbiota in ASD children. The results of this study substantially enhance our current understanding of the knowledge landscape in this field and illuminate potential avenues for future research. It is recommended that more attention be paid to the latest promising hotspots, including modulation of the gut microbiota as a potential therapy for children with ASD.

## Data Availability

All data generated or analyzed during this study are included in this published article. Other datasets used during the current study are available from the corresponding authors upon reasonable request.

## List of abbreviations

ASD: autism spectrum disorder
IF: impact factors
MGBA: microbiome-gut-brain axis
NSFC: *National Natural Science Foundation of China*
MeSH: Medical Subject Headings
NIH: National Institutes of Health
